# Comparative study on the clinical efficacy of laparoscopic cervicoisthmic cerclage and transvaginal cervical cerclage in the treatment of cervical insufficiency after hysteroscopic adhesiolysis for intrauterine adhesions: a retrospective cohort study

**DOI:** 10.3389/frph.2026.1830943

**Published:** 2026-05-25

**Authors:** Hui-Liu Fan, Xiao-Xia Wu, Zhen-Zhen Wen, Hong-Lan Wei

**Affiliations:** 1 Department of Gynaecology, Guangzhou Women and Children's Medical Center Liuzhou Hospital, Guangxi, China; 2 Department of Gynaecology, Liuzhou Maternity and Child Healthcare Hospital, Guangxi, China

**Keywords:** cervical insufficiency, intrauterine adhesions, laparoscopic abdominal cerclage, miscarriage, transvaginal cervical cerclage

## Abstract

**Introduction:**

This study compared the clinical efficacy of laparoscopic cervical cerclage (LAC) and transvaginal cervical cerclage (TVC) in treating cervical insufficiency (CI) patients after hysteroscopic adhesiolysis for intrauterine adhesions (IUA).

**Methods:**

A total of 170 eligible patients (66 in the LAC group, 104 in the TVC group) admitted from Jan 2018 to Jan 2024 were retrospectively analyzed, with LAC performed pre-pregnancy and transvaginal McDonald cerclage performed at 12–16 weeks of gestation in the TVC group. Pregnancy outcomes and surgical safety were compared between the two groups, with subgroup analysis conducted by IUA severity.

**Results:**

Results showed that the LAC group had a significantly higher term delivery rate (89.4% vs. 71.2%, *P* = 0.005) and neonatal survival rate (98.5% vs. 90.4%, *P* = 0.027), and a lower preterm birth rate before 34 weeks (9.1% vs. 20.2%, *P* = 0.036) than the TVC group. In terms of safety, the LAC group had a lower infection rate (3.0% vs. 10.6%, *P* = 0.029) and suture slippage rate (1.5% vs. 9.6%, *P* = 0.015), while the TVC group had a higher cervical laceration rate (5.8% vs. 1.5%, *P* = 0.048). The LAC group had 2 mild intraoperative complications (1.5% each), all resolved with timely treatment, whereas the TVC group had no severe intraoperative complications. The LAC group had a markedly higher cesarean section rate (98.5% vs. 26.9%, *P* < 0.001). Subgroup analysis indicated that the LAC group had a significantly higher term delivery rate in moderate-to-severe IUA patients (88.1% vs. 71.0%, *P* = 0.005), with no significant difference in mild IUA patients (91.7% vs. 71.4%, *P* > 0.05). In conclusion, LAC is superior to TVC in improving the term delivery rate and reducing late miscarriage in CI patients after IUA surgery, especially for those with moderate-to-severe IUA. TVC, with minimal invasion and preserved vaginal delivery chance, is suitable for mild IUA patients with good cervical conditions and a willingness for vaginal delivery. Individualized surgical selection is recommended in clinical practice.

## 1 Introduction

Intrauterine adhesions (IUA) are severe complications caused by endometrial injury after intrauterine operations, with an incidence rate of approximately 10%–20% (1). Among them, endometrial injury during pregnancy (intrauterine operations such as incomplete miscarriage, missed miscarriage, and artificial abortion during pregnancy) is a major factor leading to uterine injury (2). Cervical insufficiency (CI) refers to the inability of the uterine cervix to maintain its normal morphology and function in the second and third trimesters of pregnancy without uterine contractions or the onset of labor, characterized by painless and progressive dilatation and shortening of the uterine cervix, which eventually leads to preterm birth or miscarriage, with an incidence rate of 0.5%–1.0% (3, 4). If incomplete miscarriage occurs after pregnancy loss caused by CI, curettage and other intrauterine operations are the main factors leading to IUA. Therefore, some patients with CI are complicated with IUA simultaneously. Hysteroscopic adhesiolysis is the main treatment for IUA, but some patients still have CI after surgery due to cervical injury, abnormal anatomical structure, and other factors (5). CI can lead to a significant increase in the risk of late miscarriage and preterm birth. Therefore, the rate of adverse pregnancy outcomes in patients with CI after transcervical resection of adhesions (TCRA) can reach 40%–60% (6), which seriously affects reproductive health.

Cervical cerclage is the core surgical procedure for the treatment of CI, which has been proven to significantly prolong gestational age and improve obstetric outcomes (7, 8), including TVC and LAC. TVC has the advantages of simple operation and minimal invasion and is the first choice for traditional treatment. However, patients after TCRA often have problems such as cervical shortening, scarring, and decreased elasticity, which lead to a low suture position of McDonald cerclage, insufficient support, and a high failure rate (9). LAC achieves high cerclage of the uterine isthmus under laparoscopy, with stronger support and no restriction by local cervical conditions, and its application in complex CI has gradually increased in recent years (10). For CI patients with a high risk of preterm birth and miscarriage, LAC is expected to become the first choice of treatment.

However, there is a lack of evidence from large-sample, single-center retrospective studies on the comparison of the efficacy of the two surgical methods in patients with CI after TCRA.

This study collected the clinical data of patients with CI after TCRA in our hospital over the past 6 years, compared the pregnancy outcomes and safety of LAC and McDonald cerclage, aiming to provide an evidence-based basis for the selection of clinical surgical procedures and optimize the treatment strategy.

## 2 Materials and methods

### 2.1 General information

Patients with CI after TCRA who were treated in our hospital from Jan 2018 to Jan 2024 were selected and divided into the LAC group (66 cases) and the TVC group (104 cases) according to the surgical method. In the LAC group, the patients were 23–38 years old, with an average of (29.6 ± 4.2) years old; the severity of IUA: 24 cases (36.4%) of mild, 42 cases (63.6%) of moderate-to-severe; the average cervical length was (2.0 ± 0.3) cm; the history of previous miscarriage and preterm birth was 1–4 times, with an average of (2.1 ± 0.8) times. In the TVC group, the patients were 22–39 years old, with an average of (28.9 ± 4.5) years old; the severity of IUA: 42 cases (40.4%) of mild, 62 cases (59.6%) of moderate-to-severe; the average cervical length was (2.2 ± 0.4) cm; the history of previous miscarriage and preterm birth was 1–3 times, with an average of (1.9 ± 0.7) times. There was no statistically significant difference in the basic data (age, severity of IUA, cervical length, history of previous adverse pregnancy) between the two groups (*P* > 0.05), with comparability (see Table 1).

**Table 1:** General data of patients in the two groups.

Baseline characteristics	LAC group (*n* = 66)	TVC group (*n* = 104)	*P* value
Age (years)	29.6 ± 4.2	28.9 ± 4.5	>0.05
Mild IUA, *n* (%)	24 (36.4)	42 (40.4)	>0.05
Moderate to severe IUA, *n* (%)	42 (63.6)	62 (59.6)	>0.05
Cervical length (cm)	2.0 ± 0.3	2.2 ± 0.4	>0.05
History of previous miscarriage/preterm birth (times)	2.1 ± 0.8	1.9 ± 0.7	>0.05

### 2.2 Inclusion criteria

1. All patients were diagnosed with IUA by hysteroscopy and underwent TCRA. The severity grading of IUA was based on the American Fertility Society (11) (AFS) grading system. A follow-up hysteroscopy performed 3 months postoperatively indicated that the uterine cavity morphology had returned to normal (uterine cavity volume ≥5 ml, no obvious residual adhesions);
2. CI diagnostic criteria (12): (1) Medical history: ≥1 history of spontaneous miscarriage/preterm birth in the second trimester (18∼28 weeks) with painless cervical dilatation; (2) Ultrasound: cervical length <25 mm at 16∼24 weeks of gestation, with or without internal cervical os dilatation; (3) Pre-pregnancy: No resistance for an 8# cervical dilator to pass through the internal cervical os;
3. No contraindications to cervical cerclage: premature rupture of membranes, chorioamnionitis, fetal malformation, regular uterine contractions, etc.; (4) Complete clinical data and followed up to 1 month after delivery.

### 2.3 Exclusion criteria

(1) Complicated with severe internal and surgical diseases (such as hypertension, diabetes, heart disease, etc.); (2) Multiple pregnancy; (3) Excluding patients who underwent emergency cervical cerclage; (4) Excluding missed miscarriage before 12 weeks of gestation; (5) Lost to follow-up.

### 2.4 Surgical methods

#### 2.4.1 LAC group

(1) Surgical timing: Pre-pregnancy; (2) Anesthesia: Tracheal intubation general anesthesia; (3) Operation: The patient was placed in the supine position, pneumoperitoneum was established (pressure 12∼14 mmHg), 3–4 puncture points were made at the umbilicus and both sides of the lower abdomen to place laparoscopic instruments, and the uterus, bilateral adnexa, and pelvic conditions were explored; at the uterine isthmus (2–3 cm above the internal cervical os), the uterine myometrium was sutured circularly with non-absorbable suture (Mersilene tape) from anterior to posterior between the medial side of the uterine blood vessel and the outer edge of the uterine isthmus, and the needle exit point was at the upper outer side of the uterosacral ligament. Hysteroscopy was performed to exclude the penetration of the cerclage suture through the cervical canal, then the suture was tightened and knotted and fixed (5 knots) above the attachment of the uterosacral ligament under laparoscopy, and the abdomen was closed after confirming no bleeding.

#### 2.4.2 TVC group

(1) Surgical timing: 12–16 weeks of gestation; (2) Anesthesia: Spinal canal anesthesia; (3) Operation: The patient was placed in the lithotomy position, disinfected and draped, the cervix was exposed with a vaginal retractor, and the anterior lip of the cervix was gently pulled with ovum forceps; at the level of the internal cervical os and near the vaginal fornix, the cervical myometrium was sutured circularly and continuously with Mersilene tape (without penetrating the mucosal layer), the suture was tightened until the cervical canal could accommodate one fingertip, knotted and fixed at the posterior vaginal fornix, and a 1 cm suture tail was left for easy suture removal.

### 2.5 Postoperative management

#### 2.5.1 LAC group

The patients rested for 3–5 days after surgery and were given antibiotics to prevent infection. Regular prenatal examinations were performed after pregnancy to monitor cervical length and fetal development. Cesarean section was performed to terminate pregnancy at 38∼39 weeks of gestation, and the cerclage suture was not removed during the operation.

#### 2.5.2 TVC group

The patients rested for 1–3 days after surgery, were given antibiotics to prevent infection, and uterine contraction inhibitors were used as appropriate. Prenatal examinations were performed on time to monitor cervical length and fetal development. The cerclage suture was removed transvaginally at 37 weeks of gestation or before the onset of labor, vaginal delivery was attempted after evaluating cervical conditions, close monitoring was conducted during delivery, and cesarean section was performed if necessary.

### 2.6 Observation indicators

(1) Pregnancy outcomes: Term delivery rate (delivery ≥37 weeks), preterm birth rate before 34 weeks of gestation, miscarriage rate before 24 weeks of gestation, neonatal survival rate; (2) Surgical safety: Infection rate (fever, abdominal pain, abnormal vaginal secretions after surgery, confirmed infection by laboratory examination), suture slippage rate, cervical laceration rate (cervical laceration ≥ grade Ⅱ during delivery), intraoperative complications (bladder injury, bleeding, etc.); (3) Delivery mode.

### 2.7 Statistical methods

SPSS 26.0 software was used for statistical analysis. Measurement data were expressed as (x¯ ± s), and independent samples *t*-test was used for comparison between groups; count data were expressed as [*n* (%)], and *χ*^2^ test or Fisher's exact probability method was used for comparison between groups. *P* < 0.05 was considered statistically significant.

## 3 Results

### 3.1 Comparison of pregnancy outcomes between the two groups

The term delivery rate and neonatal survival rate in the LAC group were significantly higher than those in the TVC group, and the preterm birth rate before 34 weeks of gestation and miscarriage rate before 24 weeks of gestation were significantly lower than those in the TVC group, with statistically significant differences (*P* < 0.05) (see Table 2).

**Table 2:** Comparison of pregnancy outcomes between the two groups [*n* (%)].

Pregnancy outcomes	LAC group (*n* = 66)	TVC group (*n* = 104)	*χ*^2^ value	*P* value
Term delivery rate (≥37 weeks)	59 (89.4)	74 (71.2)	7.986	0.005
Preterm birth rate before 34 weeks	6 (9.1)	21 (20.2)	4.378	0.036
Miscarriage rate before 24 weeks	1 (1.5)	9 (8.7)	4.529	0.033
Neonatal survival rate	65 (98.5)	94 (90.4)	4.871	0.027

### 3.2 Comparison of surgical safety between the two groups

The infection rate and suture slippage rate in the LAC group were significantly lower than those in the TVC group, and the cervical laceration rate in the TVC group was significantly higher than that in the LAC group, with statistically significant differences. One case of lower uterine segment bleeding and 1 case of bladder injury occurred intraoperatively in the LAC group, both recovered after timely intraoperative treatment without serious adverse consequences; no severe intraoperative complications occurred in the TVC group (see Table 3).

**Table 3:** Comparison of surgical safety between the two groups [*n* (%)].

Surgical safety indicators	LAC group (*n* = 66)	TVC group (*n* = 104)	*χ*^2^ value	*P* value
Infection rate	2 (3.0)	11 (10.6)	4.783	0.029
Suture slippage rate	1 (1.5)	10 (9.6)	5.871	0.015
Cervical laceration rate	1 (1.5)	6 (5.8)	3.901	0.048
Intraoperative complications	2 (3.0)	0 (0.0)	3.105	0.078

### 3.3 Comparison of delivery modes between the two groups

Sixty-five patients in the LAC group underwent cesarean section (98.5%), and 1 patient who miscarried before 24 weeks of gestation delivered vaginally after laparoscopic cerclage removal; among 104 patients in the TVC group, 76 cases delivered vaginally (73.1%), and 28 cases converted to cesarean section due to poor cervical conditions, fetal distress, and other factors (26.9%). The difference in delivery modes between the two groups was statistically significant (*χ*^2^ = 83.542, *P* < 0.001).

### 3.4 Subgroup analysis (according to the severity of IUA)

#### 3.4.1 Moderate to severe IUA subgroup (104 cases in total: 42 cases in the LAC group, 62 cases in the TVC group)

Among patients with moderate-to-severe IUA, the term delivery rate and neonatal survival rate in the LAC group were significantly higher than those in the TVC group; the preterm birth rate before 34 weeks of gestation and miscarriage rate before 24 weeks of gestation in the LAC group were significantly lower than those in the TVC group, with statistically significant differences (*P* < 0.05).

#### 3.4.2 Mild IUA subgroup (66 cases in total: 24 cases in the LAC group, 42 cases in the TVC group)

Among patients with mild IUA, there was no statistically significant difference in the term delivery rate and neonatal survival rate between the LAC group and the TVC group; there was also no statistically significant difference in the preterm birth rate before 34 weeks of gestation and miscarriage rate before 24 weeks of gestation between the two groups (*P* > 0.05) (see Table 4).

**Table 4:** Comparison of pregnancy outcomes in subgroups with different IUA severity [*n* (%)].

Subgroup	Observation indicators	LAC group	TVC group	*χ*^2^ value	*P* value
Moderate to severe IUA LAC (*n* = 42)/TVC (*n* = 62)	Term delivery rate	37 (88.1)	44 (71.0)	7.986	0.005
	Preterm birth rate before 34 weeks	4 (9.5)	17 (27.4)	4.378	0.036
	Miscarriage rate before 24 weeks	1 (2.4)	8 (12.9)	4.529	0.033
	Neonatal survival rate	41 (97.6)	54 (87.1)	4.871	0.027
Mild IUA LAC (*n* = 24)/TVC (*n* = 42)	Term delivery rate	22 (91.7)	30 (71.4)	–	>0.05
	Preterm birth rate before 34 weeks	2 (8.3)	4 (9.5)	–	>0.05
	Miscarriage rate before 24 weeks	0 (0.0)	1 (2.4)	–	>0.05
	Neonatal survival rate	24 (100.0)	40 (95.2)	–	>0.05

## 4 Discussion

IUA, especially moderate-to-severe lesions, are the main uterine cavity factors leading to secondary infertility, recurrent miscarriage, and adverse obstetric outcomes in women of childbearing age. Their core pathological damage lies in the irreversible injury of the basal layer of the endometrium, abnormal morphology of the uterine cavity, reduction of effective volume, and accompanied by insufficient endometrial blood supply, which directly interferes with embryo implantation, placental attachment, and blood supply establishment, and is also the core mechanism of the high risk of miscarriage and preterm birth in these patients after pregnancy (13). Existing evidence-based data show that the rate of late miscarriage in patients with moderate-to-severe IUA who successfully conceived after TCRA reaches 13%–40%, and the preterm birth rate is 19%–30%, both of which are significantly higher than those in the general healthy pregnant population of childbearing age (13, 14).

In clinical diagnosis and treatment, when IUA is complicated with CI, the risk of adverse pregnancy outcomes is not a simple superposition of a single disease, but forms an amplified effect of synergistic pathogenesis, making such pregnancy a category of extremely high-risk obstetrics. The core pathology of CI is the defect and loss of function of the sphincter structure of the cervical isthmus, which cannot bear the intrauterine pressure in the second and third trimesters of pregnancy, and is prone to painless cervical dilatation and flattening in the second trimester, which then induces late miscarriage or extremely early preterm birth. Existing data confirm that the rate of late miscarriage in such patients with comorbidities rises to 40%–60%, the preterm birth rate rises to 30%–50%, and the incidence of extremely early preterm birth less than 28 weeks and recurrent second-trimester miscarriage is significantly higher than that in patients with a single disease (1). A special retrospective study by Li et al. (15) on IUA complicated with CI also confirmed that the incidence of adverse pregnancy outcomes in the comorbidity group was much higher than that in the simple IUA group or simple CI group, with a statistically significant difference, suggesting that clinical practice should abandon the fragmented diagnosis and treatment idea of a single disease and adopt targeted combined intervention measures.

Cervical cerclage is a currently recognized effective intervention for late miscarriage and preterm birth caused by CI, and the selection of an appropriate cervical cerclage method is crucial to improving the pregnancy outcome of patients with CI after TCRA.

The results of this study showed that the term delivery rate in the LAC group (89.4%) was significantly higher than that in the TVC group (71.2%), and the preterm birth rate before 34 weeks of gestation (9.1%) and miscarriage rate before 24 weeks of gestation (1.5%) were significantly lower than those in the TVC group (20.2%, 8.7%), which was consistent with the research conclusions of Li et al. (7) and Smith et al. (16). The core advantage of LAC is the higher cerclage position (uterine isthmus, 2–3 cm above the internal cervical os), which can directly support the internal uterine os, effectively block the traction of cervical dilatation of the lower uterine segment on the cervix in the second and third trimesters of pregnancy, avoid premature opening of the internal cervical os, thus significantly reducing the risk of preterm birth (10). In addition, LAC is mostly performed before pregnancy or in the first trimester, when the uterine volume is small and the operation space is sufficient, which can realize accurate suture of the uterine isthmus without being affected by cervical scars and shortening. However, TVC needs to be performed in the second trimester, when the cervix has begun physiological changes, and the cervical elasticity of some patients after IUA surgery is poor, so it is difficult to find a stable tissue support during suture, leading to poor cerclage effect (17).

In terms of safety, the infection rate (3.0%) and suture slippage rate (1.5%) in the LAC group were significantly lower than those in the TVC group (10.6%, 9.6%), because the cerclage position of LAC is located at the uterine isthmus, at the level of the internal cervical os, which is more in line with the characteristics of anatomical physiology, and the suture is fixed in the uterine myometrium with a firm position and not easy to slip (18). Therefore, LAC can better maintain cervical length than TVC (18). At the same time, LAC can avoid retrograde infection caused by fetal membranes entering the dilated internal cervical os or loss of cervical mucus plug due to cervical shortening (19), reduce premature rupture of membranes, and reduce the incidence of miscarriage/preterm birth. However, the suture of TVC is exposed to the vaginal environment, which is easily affected by secretions, sexual life, and other factors, increasing the risk of infection and slippage. In particular, the cervical mucosal repair of some patients after TCRA is poor, and infection is more likely to lead to cerclage failure (20). However, LAC has a certain risk of intraoperative complications (the incidence rate in this study was 3.0%), mainly bladder injury and surrounding tissue bleeding, which are related to the surgical skills of laparoscopic operation and the complex anatomical position of the uterine isthmus, and need to be performed by experienced physicians to reduce the risk of complications (12).

In terms of delivery methods, the LAC group almost requires cesarean section, which is consistent with the research results of Seo et al. (21). The study reported that when laparoscopic cervical isthmus cerclage was performed on patients with refractory cervical dysfunction, the fetal survival rate reached 93%, and the cesarean section rate also reached 93%. This is because the suture of LAC is located in the isthmus of the uterus, which cannot be removed vaginally, and the elasticity of the cervix is poor after cerclage, making it difficult for the cervix to dilate during vaginal delivery, which can easily lead to serious complications such as cervical laceration and uterine rupture (10). Therefore, it is necessary to choose cesarean section. Although this can ensure the safety of both mother and baby during delivery, it also increases the risk of complications related to cesarean section, such as bleeding during delivery, uterine rupture during another pregnancy, placental implantation, scarred uterus, etc. (22, 23), leading to an increased risk of short-term and long-term poor prognosis for LAC pregnant women. In addition, the total cost of cerclage and delivery for LAC pregnant women is significantly higher than that for TVC pregnant women. From a health economics perspective, LAC increases the economic burden on pregnant women (24, 25). 73.1% of patients in the TVC group can undergo vaginal delivery, avoiding trauma related to cesarean section and reducing the occurrence of some complications, which is more in line with patients’ needs for natural delivery (9).

Therefore, when selecting LAC for patients, clinical physicians should fully inform them of the necessity and potential risks of cesarean section, and weigh the benefits of improving pregnancy outcomes against the drawbacks of surgical delivery. In contrast, the TVC group has a higher vaginal delivery rate, which may be more advantageous for patients who wish to have vaginal delivery and whose cervical conditions are suitable for vaginal cerclage. LAC is preferred for patients with a history of failed vaginal cerclage or complex uterine conditions (such as after TCRA).

Subgroup analysis showed that the advantages of the LAC group were more significant in patients with moderate-to-severe IUA after surgery, because the thin endometrium, insufficient uterine cavity volume, and abnormal placental attachment caused by moderate-to-severe IUA will further aggravate the problems of poor pregnancy stability and imbalance of intrauterine environment, induce uterine contractions, and greatly increase the incidence of adverse pregnancy outcomes. In addition, patients with moderate-to-severe IUA have more severe cervical injury, more obvious scarring and shortening, and insufficient support during TVC suture, resulting in a higher failure rate; while patients with mild IUA have better cervical conditions and ideal uterine cavity recovery, TVC can achieve pregnancy outcomes similar to LAC without cesarean section, which is more clinically applicable (17).

One major limitation of this study is the difference in the timing of intervention between the two groups: LAC was performed pre-pregnancy, while TVC was performed in the second trimester. This discrepancy may act as a potential confounder, as pre-pregnancy cerclage may be associated with better preoperative preparation and lower risk of intraoperative fetal injury, which could influence the comparison of pregnancy outcomes. Future prospective, randomized controlled trials with uniform intervention timing are needed to validate our findings.

Both surgical methods have certain limitations: LAC has large surgical trauma and high cost, and cesarean section is necessary after surgery, which increases maternal and infant trauma; TVC has a high rate of miscarriage and preterm birth in patients with moderate-to-severe IUA. Clinical decision-making needs to comprehensively consider the severity of IUA, cervical anatomical conditions, previous pregnancy history, and patients’ wishes: patients with moderate-to-severe IUA after surgery, cervical shortening (<2.5 cm), severe scarring, or previous TVC failure should prefer LAC; patients with mild IUA after surgery, good cervical conditions, and the willingness to deliver vaginally can choose TVC. This study is a single-center retrospective study with certain limitations: the sample size is relatively limited, and there may be selection bias; the long-term prognosis of patients (such as recurrent IUA after miscarriage, subsequent pregnancy outcomes, cervical function recovery, etc.) was not followed up. In the future, multi-center, prospective randomized controlled studies need to be carried out to further verify the long-term efficacy and safety of the two surgical methods.

## 5 Conclusion

Among patients with CI after TCRA, pre-pregnancy LAC is significantly superior to TVC in terms of term delivery rate, preterm birth rate, late miscarriage rate, and neonatal survival rate, and has lower infection rate and suture slippage rate. It is the first choice for patients with moderate-to-severe IUA after surgery, cervical shortening, or previous TVC failure; TVC has the advantages of minimal invasive trauma and reserving the chance of vaginal delivery, which is suitable for patients with mild IUA after surgery and good cervical conditions. Clinical practice needs to combine the severity of patients’ IUA, cervical anatomical characteristics, and personal wishes to select surgical procedures individually to optimize pregnancy outcomes.

## Data Availability

The original contributions presented in the study are included in the article/Supplementary Material, further inquiries can be directed to the corresponding author.
